# Healthcare system resilience in Bangladesh and Haiti in times of global changes (climate-related events, migration and Covid-19): an interdisciplinary mixed method research protocol

**DOI:** 10.1186/s12913-021-07294-3

**Published:** 2022-03-15

**Authors:** Lucie Clech, Sofia Meister, Maeva Belloiseau, Tarik Benmarhnia, Emmanuel Bonnet, Alain Casseus, Patrick Cloos, Christian Dagenais, Manuela De Allegri, Annabel Desgrées du Loû, Lucas Franceschin, Jean-Marc Goudet, Daniel Henrys, Dominique Mathon, Mowtushi Matin, Ludovic Queuille, Malabika Sarker, Charlotte Paillard Turenne, Valéry Ridde

**Affiliations:** 1 Centre Population et Développement (Ceped), Université de Paris, IRD, INSERM, Ceped, F-75006 Paris, France; 2 Herbert Wertheim School of Public Health & Scripps Institution of Oceanography University of California, San Diego, CA USA; 3 IRD, UMR 215 Prodig, 5, cours des Humanités, Cedex, F-93 322 Aubervilliers, France; 4 Zanmi Lasante, Cange, Haiti; 5 École supérieure d’infotronique d’Haïti, Port-au-Prince, Haiti; 6 Département de médecine sociale et préventive, École de santé publique, École de travail social, Centre de recherche en santé publique (CRESP), Université de Montréal, Québec, Canada; 7 Département de psychologie, Université de Montréal, Québec, Canada; 8 Heidelberg Institute of Global Health, University Hospital and Medical Faculty, University of Heidelberg, Heidelberg, Germany; 9 fellow of the French Collaborative Insitute on Migration, Paris, France; 10 Université du Québec, Montréal, Québec Canada; 11 BRAC James P Grant School of Public Health, BRAC University, 68 Shahid Tajuddin Ahmed Sharani, Mohakhali, Dhaka, 1212 Bangladesh; 12 World Bank, Washington, DC USA

**Keywords:** Healthcare systems resilience, Global change, Climate change, Migration, Covid-19, Mixed-methods

## Abstract

**Background:**

Since climate change, pandemics and population mobility are challenging healthcare systems, an empirical and integrative research to studying and help improving the health systems resilience is needed. We present an interdisciplinary and mixed-methods research protocol, ClimHB, focusing on vulnerable localities in Bangladesh and Haiti, two countries highly sensitive to global changes. We develop a protocol studying the resilience of the healthcare system at multiple levels in the context of climate change and variability, population mobility and the Covid-19 pandemic, both from an institutional and community perspective.

**Methods:**

The conceptual framework designed is based on a combination of Levesque’s Health Access Framework and the Foreign, Commonwealth and Development Office’s Resilience Framework to address both outputs and the processes of resilience of healthcare systems. It uses a mixed-method sequential exploratory research design combining multi-sites and longitudinal approaches. Forty clusters spread over four sites will be studied to understand the importance of context, involving more than 40 healthcare service providers and 2000 households to be surveyed. We will collect primary data through questionnaires, in-depth and semi-structured interviews, focus groups and participatory filming. We will also use secondary data on environmental events sensitive to climate change and potential health risks, healthcare providers’ functioning and organisation. Statistical analyses will include event-history analyses, development of composite indices, multilevel modelling and spatial analyses.

**Discussion:**

This research will generate inter-disciplinary evidence and thus, through knowledge transfer activities, contribute to research on low and middle-income countries (LMIC) health systems and global changes and will better inform decision-makers and populations.

**Supplementary Information:**

The online version contains supplementary material available at 10.1186/s12913-021-07294-3.

## Background

Contemporary pandemics, mobilities and climate change have highlighted more than ever the need to develop an empirical, integrative, applied approach for documenting the resilience of healthcare systems beyond concepts in global health [1]. This situation is all the more dramatic for low and middle-income countries (LMIC) as the costs of these shocks and future, unforeseen emergencies will drain already scarce resources and accentuate existing inequities. Based on conceptual analysis and other empirical papers [2, 3], we define health systems resilience as: the constituents’ abilities of a health system facing destabilising experiences, events or shocks (contingent or expected, sudden or insidious, internal or external) to adapt and transform in order to maintain and/or improve access (for all) to comprehensive, relevant and quality healthcare and services without pushing patients into poverty.

Health systems resilience (HSR) is at the core of current preoccupations. The 2020 Lancet Countdown report on health and climate changes recommend to integrate climate change and the COVID-19 crisis responses, in a way that addresses inequality directly [4]. Globally, 400 million people have no access to essential health services [5]. Achieving universal health coverage (UHC), including financial risk protection, access to quality essential healthcare services and access to safe, effective, quality, and affordable essential medicines and vaccines for all is part of the sustainable Development Goal (SDG) 3.8 for 2030. “For all” is essential in this declaration, as a significant proportion of the population, such as migrants, do face distinctive vulnerabilities to poor health [6] and poor healthcare access [7].

Various kind of emergencies are expected to continue happening, but a more resilient and robust healthcare system can minimize their impacts, which is within reach of all countries, even the poorest [8]. Because low- and middle-income countries (LMIC) and their populations face numerous social vulnerabilities and are already fragile in terms of health coverage and health structures; the necessity to document the resilience of national healthcare systems is even more compelling. There is an urgent need to build empirical evidence on the resilience of health systems and the strategies stakeholders organize to respond to these crises in order to thoroughly inform decision-makers to better anticipate.

Despite health system resilience being a widely researched topic, this concept lacks of conceptual maturity, needs clarification and empirical validation [2]. In this context, we present the design of an interdisciplinary research project (ClimHB project) focusing on health systems resilience through the example of vulnerable localities in Haiti and Bangladesh. These two countries are highly sensitive to climate change and other global and local risks. These changes and risks are pushing out people from their communities, or trapping them when unable to move away [9] thus influencing populations mobility and immobility. Both countries are experiencing global changes through the exacerbation of disruptions caused by climate-related events, (im)mobility and the Covid-19 pandemic. In order to understand how local contexts, influence and interact with patterns of resilience and population health, we use a multi-sites approach, to integrate a variety of local contexts and vulnerabilities, because context matters [10–12]. This project aims to answer two interrelated questions in the context of climate-induced environmental change (CIEC), population (im)mobilities, Covid-19 pandemic and other possible changes, events and risks affecting the population and the healthcare systems:

1. What is the relative resilience of local health service providers in various settings vulnerable to climate change and (im)mobility in Haiti and Bangladesh?
2. What are the patterns and distribution of health status and access to healthcare services of the (im)mobile populations in these various settings in Bangladesh and Haiti?

In the first section of this paper, we present our conceptual framework, study sites and methodologies. In the second section, we discuss the challenges and innovations of this approach and protocol.

## Methods/design

### Conceptual framework

**Fig. 1:**
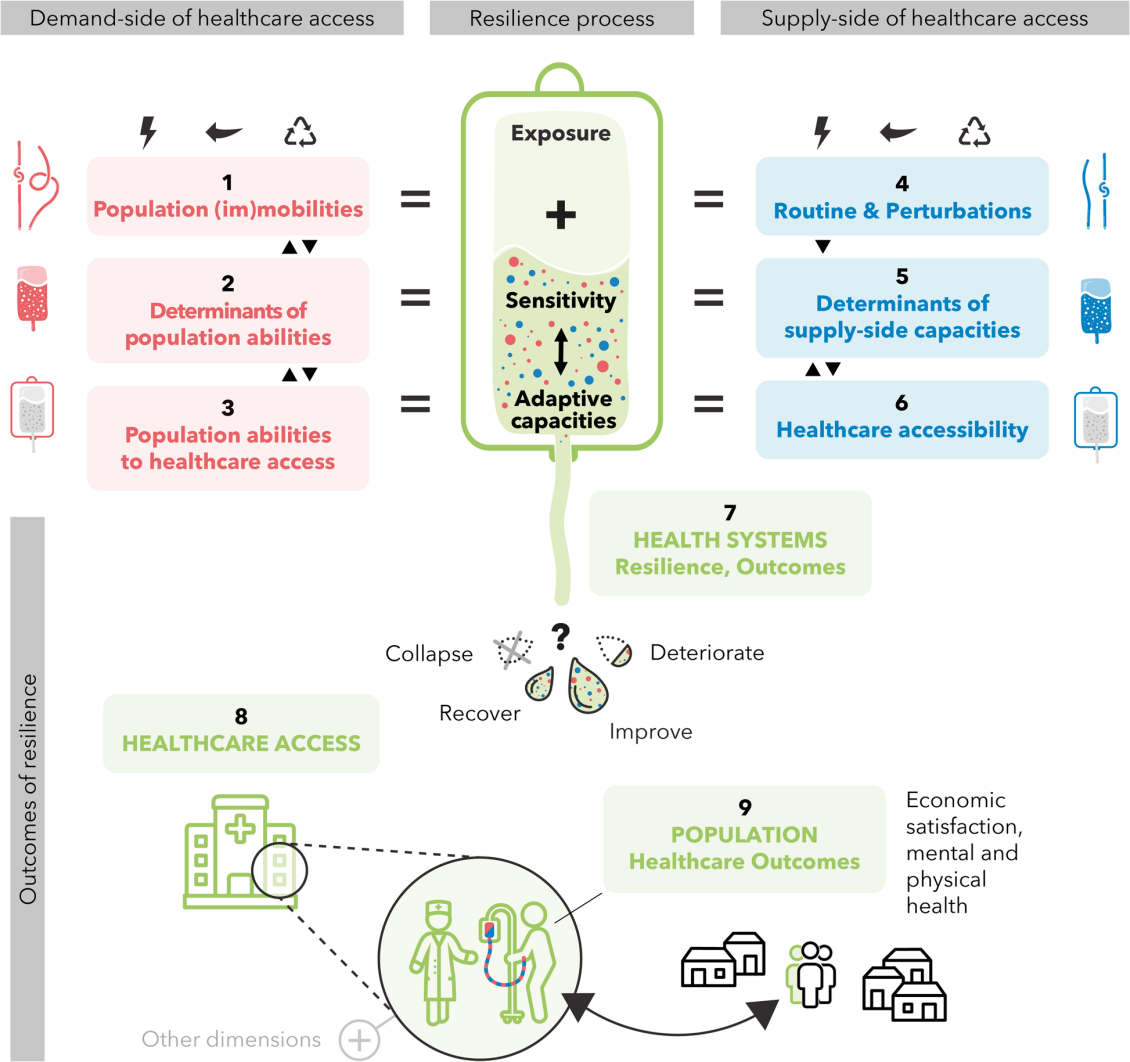
Conceptual framework. Conceptual framework explanatory notes: ClimHB integrative framework of health system resilience is built on an adapted combination of the Levesque framework on healthcare access [15] and the DFID framework [16] on the resilience process. It frames health system resilience outcomes through the lens of healthcare access, seen here as an essential outcome of the health system resilience and as one of the determinants of population health. The strengths of this framework are the inclusion of the population as a component of the health system (demand side), side by side and interacting with the health services and providers (supply side), and the inclusion of the resilience process (exposure, sensitivity, adaptive capacities) and its outcomes (healthcare access and population health). We have chosen to include both demand and supply in this framework to include all dimensions of the health system and access to healthcare. We choose the transfusion bag (in green in the centre) as a health metaphor to graphically represent the supply and demand interactions. The different components (tube, fluid and bag in the strict sense) are explained on both the right and left sides. We pictured in mirror 1., 2., 3. (demand side) and 4., 5., 6. (supply side) because it shares some similarities in its integration in the framework: 1. and 4. are equivalent of exposure, while 2. and 5. are about sensitivity and 3. and 6. are about the adaptative capacities of the system. Exposure concerns “the presence of people; livelihoods; species or ecosystems; environmental functions, services, and resources; infrastructure; or economic, social, or cultural assets in places and settings that could be adversely affected” [17] and can be measured as an “assessment of the magnitude or/and frequency” of disturbing events [16]. Sensitivity is the degree to which a system will/might be affected by, or respond to, a disturbing event [16], for example, by climate change or variability [17]. Adaptive capacities and abilities are determined by the abilities of systems, institutions, humans and other organisms to adjust to potential damage, to take advantage of opportunities, or to respond to consequences [17], allowing actors “to anticipate, plan, react to and learn from events” [16]. The system’s capacity to deal with all kinds of disturbance depends on exposure, sensitivity and adaptive capacities, with adaptive capacities interacting with the type and degree of exposure and sensitivity. To better understand the figure, the following section aims to present and explain the different components. Population (im)mobilities refer to a) all mobilities from daily movements to displacement or long-term migration and b) all situations of immobility, undergone or voluntary. Mobility is represented by the red tube, while immobility is represented by the red tube with the knot. The three symbols above refer to all events disrupting the determinants of population abilities and healthcare access abilities, such as sudden shocks (or sudden events), stresses (long-term trends), challenges and chronic tensions affecting the supply and/or the demand sides. Because the ClimHB project focuses on the context of climate change and population mobilities and immobilities, which might influence other determinants, (im)mobility was highlighted from the list of determinants from Levesque and included in (1.) with a focus on migratory status, in interaction with shocks and events. Due to the numerous categories of mobilities that might interact with each other’s, “(im)mobility” was also kept in the determinants (2.), with a focus on physical capacities (ability to move/stay). Determinants of population abilities include all socio-economic characteristics of the individuals and their communities, from empowerment to various capitals and health literacy. The red fluid represents it. Population abilities to healthcare access encompass the five dimensions of access capturing the demand-side determinants (cited in 2.): the abilities to perceive, to seek, to reach, to pay and to engage. This is represented by the red bag (tube and fluid). Population (im)mobilities, population abilities and population abilities to healthcare access (1., 2. and 3.) are presented linearly because of 2D but are interconnected in 3D; i.e. population abilities to healthcare access might be influenced and might influence both population abilities and population (im)mobilities. Events may (or not) impact mobilities and population abilities. Routines and perturbations involve all events disrupting or all events, that could, but are not disrupting (represented by the three symbols above) the supply sides’s normal functioning, routines and habits (healthcare services and providers). This includes sudden shocks, stresses and challenges and chronic tensions, which might originate from climate changes and population (im)mobilities, among other events. The pictured straight blue tube represents the usual functioning (routine) of healthcare access from the supply side, and the second blue tube (with a knot) represents routine perturbations. Determinants of supply-side capacities include all characteristics impacted by or resulting (or not) from the change following the events, such as the building blocks [18], the hardware and software of health system [19] or from information screening to transparency outreach as defined by Levesque [15]. The blue fluid represents it. Healthcare accessibility encompasses the five dimensions of accessibility of services capturing the supply-side determinants (the health system dimensions in 5.): approachability, acceptability, availability and accommodation, affordability and appropriateness. Routines and perturbations, health system dimensions and healthcare accessibility (4., 5., 6.) are also presented linearly due to 2D but are interconnected in 3D; routines and perturbations are impacting determinants of supply-side capacities, which interact with healthcare accessibility. Resilience (7.), pictured in green, is the combination of the demand side (red) and the supply side (blue) of healthcare access across exposure, sensitivity and adaptive capacities (pictured in green). Depending on these three components, the healthcare system resilience can be characterized by assessing its outcome: healthcare access (8.), which might collapse, recover, deteriorate or improve compared to the usual trend (state and dynamic, without effects from disturbing events). Here, population health outcomes (9.) are considered the ultimate outcome of the resilience of the system and as an important result of healthcare access. Other determinants of healthcare access are represented in grey

While the World Health Organization (WHO) is calling governments to improve health systems resilience, this concept is yet to be clarified [2]. Our choice of definition includes all types of destabilising experiences affecting systems such as events (shocks, crisis), (everyday) changes and risks and encompasses both the institutional and the patient level. In this context, we propose our conceptual framework (Fig. 1) to systematically analyse the resilience processes at both provider and population levels and their effects on healthcare access and utilisation. In other words, we tried to develop a framework to understand both the resilience processes and the output of the resilience (health access). Various healthcare-use conceptual models have been proposed [13, 14]. Yet, they have in common that access to care is “the opportunity to reach and obtain appropriate healthcare services in situations of perceived need for care” [15]. The Levesque’s Conceptual Framework of Access to Health [15] model stands out as it encompasses both healthcare service providers and population dimensions with five dimensions related to the institutional level (approachability, acceptability, availability and accommodation, affordability and appropriateness) and five capacities related to individuals or households (abilities to perceive, to seek, to reach to pay and to engage). We extend this framework with a conceptual resilience approach developed by the Foreign, Commonwealth and Development Office (FCDO, formerly UK Department for International Development- DFID). According to the FCDO, most resilience definitions share four common elements: i) context; ii) disturbance; iii) capacity to deal with disturbance; and iv) reaction to disturbance which appears relevant in our context of climate-related and other events and changes in Haiti and Bangladesh. FCDO has developed a framework for integrating resilience into their work on climate change and conflict prevention based on these four elements, because “it can be used to examine different kinds of resilience and help determine the level of resilience that exists” [16].

We base our integrated analytical framework on both the FCDO conceptual proposal [16] and the importance of interactions between the health system and the population in achieving access to care, as Levesque et al. suggested [15] (Fig. 1). Consequently, we focus on both the process and outputs of local health system resilience in the context of global and local changes, including 1) environmental risks such as climatic-related events and pandemics such as Covid-19, 2) (im)mobilities and migration as socio-demographic changes and 3) any other global and local events, changes and risks that could affect healthcare systems at institutional and/or population levels.

#### Study setting/context

According to the Global Climate Risk Index in date, Bangladesh was ranked 7th and Haiti 3rd on the Long-Term Climate Risk Index [20]. Both countries are prone to disasters due to their geographical location and environmental characteristics. They are subject to hydro-meteorological disasters; especially cyclones, heavy rainfalls and flooding. Besides, Haiti is facing geophysical hazards. Vulnerability to climate change is also related to the combined consequences of other structural and institutional factors: deep social inequality, high poverty rate, poor public health infrastructure and accessibility, land use and access, high population density (Bangladesh), rapid urbanisation, civil unrest and violence (Haiti) [21–23]. The context in Haiti is that of severe socioeconomic deterioration, denunciation of corruption, long lasting political crisis and recent constitutional crisis. This has resulted in lockdowns, massive social unrest and demonstrations. Since 2018, Haiti has been experiencing a significant increase in human rights violations and abuses, crime and massacres, kidnappings, restrictions on freedom of expression and freedom of the press, continuing impunity and aggravated food insecurity [24, 25]. This serious socio-political instability has led to the closure of some care facilities [26]. Mobility, whether climate-induced or not [27], could put an additional strain on healthcare systems. Because population mobility and migration have multiple causes and have a complex relationship with climate change, scientists are cautious about linking mobility and migration to climate change [28]. However, some climate-related events have been shown to directly or indirectly influence mobility and migration decisions [29]. This nuance being stated, mobility and migration affect the healthcare system at two levels: at the organisational level, for example with a structure, services and workforce that are not adapted to the patients and at the population level with an increased vulnerability of the population. Both health system performances are poor: Bangladesh is ranked 88th and Haiti 148th out of 191 countries in 2000 by the WHO. When considering the UHC effective coverage index in 2019,Bangladesh is in the 6–7 decile and Haiti is in the lowest decile [30].

#### Sites selection

**Fig. 2:**
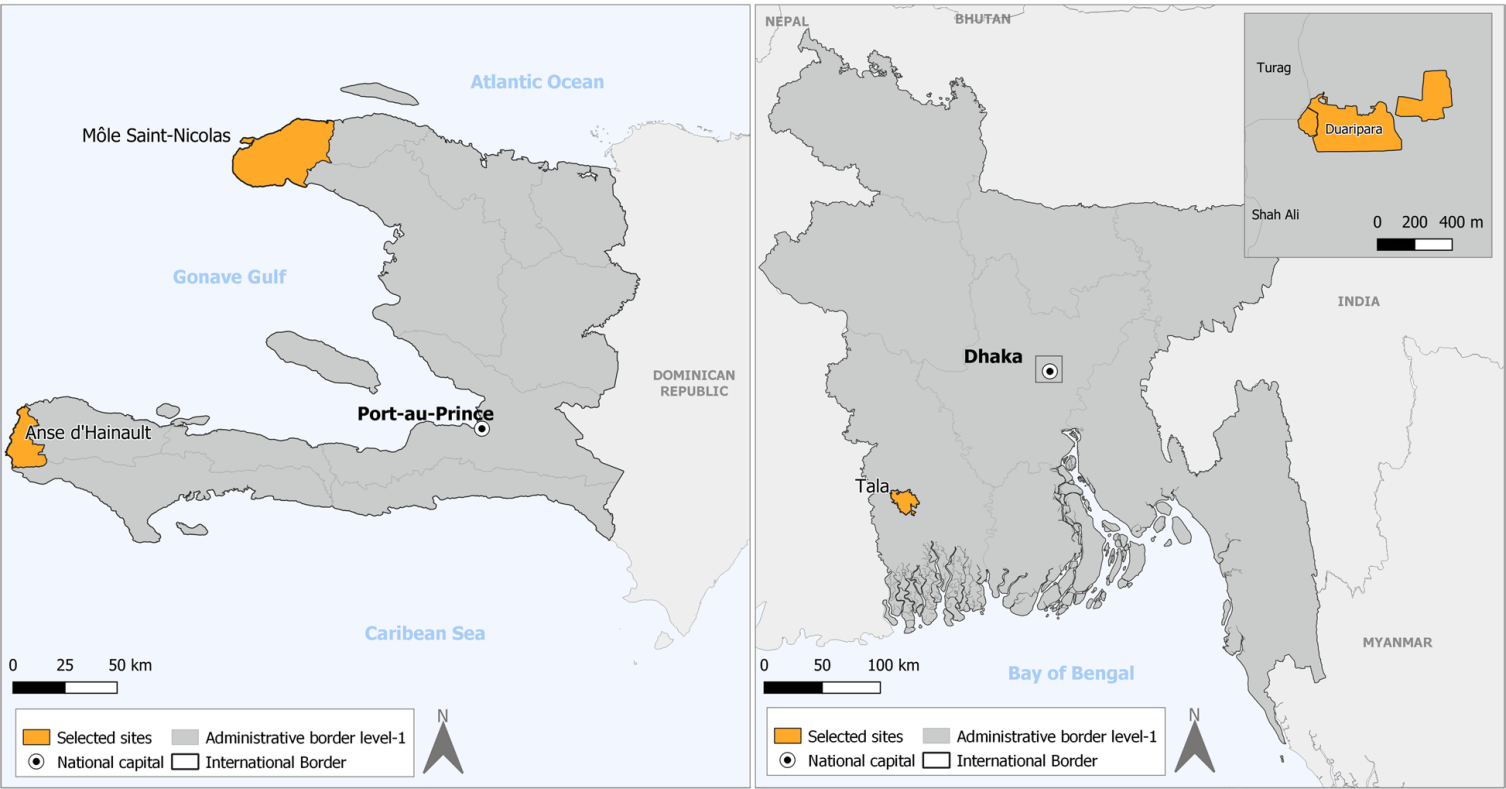
Study sites in Haiti and Bangladesh. Study sites are in orange. Maps are original

Exploratory key informant interviews and reviewed secondary data resources were conducted to select two sites in each country, based on climate-induced vulnerabilities, mobility patterns and availability of health system secondary data for the past decade when possible. Sites boundaries are the ones defined by local health system units. We selected for their local context two rural coastal Haitian sites (Anse d’Hainault and Môle St Nicolas) and one rural (Tala) and one urban (Duaripara slum) Bangladeshi sites (Fig. 2, Table 1).

**Table 1:** Study sites

	Haiti		Bangladesh	
Sites	Môle St Nicolas	Anse d’Hainault	Duaripara Slum in Dhaka	Tala
**Type of site**	Rural	Rural	Urban	Rural
**Climate-induced risks**	Droughts, floods (alluvial plain), storms, erosion	Marine submersion, floods on coastal plains, storms, erosion, land slides	Flooding, standing water, fires, and storms	Flooding, waterlogging
**Migration**	Migration due to droughts	Migration due to property loss and damage/cyclones	Rural-urban migration due to riverbank erosion/ property loss and damage due to cyclones	Out-migration of male laborers
**HSP choice criterion**	5 consultations per day		30 consultations per day	
	and represent the diversity of levels (first and second), geographical situations (urban and rural) and types (private, for-profit, private non-profit, public)			
**Clusters radius**	1-3 km	1-3 km	1 km	1 km
**Number of HSPs and clusters**	10	10	10	10
**Number of households per cluster**	500	500	500	500

Regarding migration and mobility in Bangladesh in 2020, 88,000 people living in displacement (IDPs) were registered due to disasters and 427,000 IDPs due to conflict and violence, which, for most, are protracted cases from the partitioning and independence of the country, were recorded [31]. As of 31 December 2019, 4,086,000 new displacements due to disasters were recorded, many in the form of life-saving pre-emptive evacuations, and 520 new displacements due to Intercommunal violence against Ahmadi Muslims in Rangpur division and political violence following elections [31].

Tala upazila (or Tala subdistrict) is located in the south-western coastal region of Bangladesh, in the Satkhira district of Khulna division, which is one of the most disaster-prone areas of the country, especially for flooding and waterlogging [32, 33]. Tala has at least 30,000 inhabitants, 15 to 20 community clinics and five sub-districts hospitals. The primary source of income is agriculture. Men, pushed out by lands waterlogging, migrate out for labour, while women, children and elderly, remain behind with poor access to sanitation and drinking water. Pregnant women often suffer from reproductive health issues because they cannot access healthcare [34].

Duaripara slum is located in ward 6 of Dhaka North City Corporation. Most households are extremely poor according to BRAC Urban Development Program (UDP). Its population increased recently when residents from the nearby Bhola slum moved here after they were evicted. Most of the Bhola slum residents migrated from Bhola Island after losing their land due to riverbank erosion or property loss and damage due to cyclones [35]. Healthcare is mostly limited to over-the-counter treatment at pharmacies; residents go to government and private hospitals, which are far, only during serious illnesses. 24.7% of the residents work in a garments factory followed by 15.6% of residents who work as day labourers and other significant occupations are rickshaw-pulling and domestic work [36]. All houses have electricity, although non officially, and piped water is shared by around ten households. The slum settlement area is built on wetlands. Flooding, standing water, fires, and storms, especially during the monsoon season, are frequent in the area.

In Haiti, many people displaced by the 2010 earthquake and hurricanes such as Matthew in 2016, are still living in displacement [37]. As of 31 December 2019, the total number of people living in displacement (IDPs) includes 51,000 IDPs due to disasters and 2100 IDPs due to conflict or violence [37]. For 2019, 1200 new displacements due to disasters and 2100 due to conflict and violence were recorded in Haiti [37].

Anse d’Hainault arrondissement is located in the Grand’Anse Department which has been affected by Hurricane Matthew in 2016. It has an estimated population of 98,522 inhabitants (IHSI estimates for 2015), with an extreme poverty rate of around 35% and counts 11 health facilities: six dispensaries, two health centres without beds, one with beds and two community hospitals. Coastal plains are subject to marine submersion as well as floods. Essential services like access to water or electricity are deficient. In rural areas, as in Mole St Nicolas, agriculture is the primary source of income.

Môle Saint-Nicolas arrondissement has an estimated population of 245,590 inhabitants (IHSI estimates for 2015) and is considered one of Haiti’s poorest departments, with an extreme poverty rate above 40%. It counts 36 health facilities: 30 dispensaries, two health centres without beds, three with beds and one community hospital.

#### Data collection methods

**Fig. 3:**
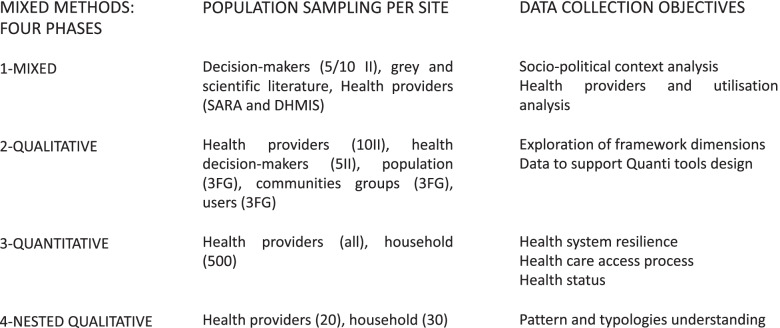
Framework design

We use a mixed-methods sequential exploratory research design [38] with a four-phase organisation (Fig. 3: framework design).

The first exploratory (contextual) phase consists of a contextual analysis to describe, political, social, economic, and health factors and their relationships, both at the national and local levels, through 1) a compilation of local monographs describing the four sites’ socio-political organisation, the history of the communities and their livelihoods, local healthcare system and demographic trends, 2) public health and public health reforms and migration policies at the national level, and any other experiences that have an impact on the local health system and population, through interviews, document analysis, and public service records and archives. In addition, a quantitative analysis of secondary data from routinely collected data (DHMIS) and SARA surveys, combined with the development of indicators, will be conducted to better understand the impacts of risks, events and climate change over the past decade on healthcare service providers (HSPs) at the four sites. These analyses are intended to allow for longitudinal and transversal analyses.

For the second phase, we use a qualitative methodology with a mixed inductive-deductive approach. This phase aims to map out the variety of disruptions and disturbances faced by HSPs and the populations and identify the diverse coping strategies implemented to understand the factors and stages in the process of resilience in the four sites. Mobility and migration will be part of the discussion on coping strategies of the population. In-depth and semi-directed interviews with national (*n* = 2) and regional/local (*n* = 1 + 2) health decisions makers and with the heads of the 10 selected healthcare service providers within each site, and focus group discussions with inhabitants of the community (*n* = 3), community-based groups (n = 3) and healthcare users (n = 3) will be organised within each site too. Those numbers will be adapted following a purposeful sampling strategy, based on criteria (age, sex, socio-economic status, being a patient, place of living) and according to the results of pilots and each previous phase [39]. Finally, a mental health research arm, whose specific protocol will be described in another publication at a later stage, will be carried out to further investigate issues pertaining to mental health and trauma in the face of extreme environmental events. For this embedded study, qualitative data will be collected to complement our quantitative data, at a different timing than phase 2.

During the third phase, all HSP health staff and a sample of household members from the communities around the 10 selected HSPs will be interviewed to complete questionnaires (*n* = 500 households per site- see below for sampling strategy and eligibility criteria). This quantitative phase aims to quantify the (perceived) impacts of identified disruptive events, changes and risks on the healthcare providers, healthcare access and population health status, based on the results from phase 2. Specifically, the household questionnaire includes different modules in two sections, a household and a respondent section. The respondent section includes socio-demographic information, health status (acute and chronic diseases), reproductive health, maternal health and family planning, mental health and trauma, empowerment, history of health and history of mobility, social capital, livelihood security and community perception, and a module on healthcare access. The household section includes household socio-demographic and economic information. The HSP questionnaire includes questions on five supply-side determinants on healthcare access [15] in connection with the different SARA surveys available in both countries. It will describe the situation and assess, if possible, the impacts of disruptive events on local healthcare service providers. Interview guides (phase 2) and questionnaires (phase 3) are presented in the supplementary materials.

The fourth phase aims to document further and better understand the typologies and strategies that will have emerged from the two previous phases. During this qualitative phase, a nested sample of health professionals (*n* = 20) and household members (*n* = 30 households) will be interviewed.

#### Eligibility criteria and sampling strategy

We will select ten HSPs per site (20 per country) to represent the diversity of levels (first and second), geographical situations (urban and rural) and types (private, for-profit, private non-profit, public) of each site. We will consider only HSPs with a minimum attendance level: five consultations per day in Haiti and 30 in Bangladesh.

For the phase 2, we will identify key informants who will be able to provide specific information on our study topic [39] and will be selected to have a wide range of understanding of the phenomenon. Regarding the decision-makers (*n* = 5), they will be interviewed at the local (*n* = 2), regional (*n* = 1) and also national levels (n = 2).

Community inhabitants (mostly non-users of healthcare), community groups (women’s groups, trade unions, community health workers, etc.) and users of healthcare services (15 patients will be randomly selected from three of the 10 healthcare service providers selected above, while taking account of gender and residence distance from HSP), will be enrolled for focus groups participation according to their knowledge and experience. Community leaders, local NGOs and healthcare facility managers will assist us in selecting these individuals.

To implement phase 3, 500 households per site (1000/country), spread over 10 clusters corresponding to the 10 HSPS will be selected in each site. Clusters will coincide with circles with a kilometre radius in Bangladesh and a 1-to-3 km radius in Haiti around the previously selected HSPs. In each circle, we will identify households through photo interpretation. Then, we will select a spatialized random sample of 50 households.

The household survey will be conducted to select households that are

- with recent (3 weeks) contact with an HSP for child immunization and/or
- with a recent episode of illness (3 weeks) for children and adults or/and
- with a birth in the last 12 months or/and
- with a pregnant woman

Whenever possible, we will interview all adults who slept in the home the previous night. Questions about children and under 18 who also slept in the home the previous night will be asked of the child’s primary caregivers. A minimum of 100 adults per cluster will be interviewed. The number of households visited will be adjusted accordingly but should be around 50. Finally, during Phase 3, all health staff of the 10 local HSPs will be administered the HSP questionnaire.

For the qualitative phase 4, a nested sub-sample of households and HSP will be selected from among those who responded to the Healthcare Service Provider and Household Questionnaires in Phase 3, for in-depth qualitative interviews with HSPs (*n* = 16) and users and non-users (*n* = 40). These numbers are approximate as they will be determined based on the preliminary results of the quantitative analyses.

#### Sample size

We will recruit a sample of 500 households per site (*N* = 4 × 500). Sample size calculations are based on the main outcome variable, scale for self-rated health [40]. Our targeted sample of 2000 households would yield a minimal detectable size of 12% at 80% power for a type I error (α) of 5% [41] using a linear regression. Such effect size is comparable or below to estimates reported in the literature [42, 43].

#### Data management

A research data management plan will be created to help researchers manage the data as part of their research activity or project as well as to facilitate the sharing of data at the end of the project at https://dataverse.ird.fr.

#### Analytical approach

##### Qualitative data

All interviews and focus group discussions will be digitally recorded, transcribed and coded using NVivo software. A code tree will be determined based on the conceptual framework. New codes may be added if new dimensions emerge during the process. We will perform two types of analyses: a framework analysis [44] to highlight empirical data and a more inductive analysis to allow a grounded understanding beyond our initial conceptual framework.

##### Quantitative data

All data will be collected on tablets and immediately transmitted to a secure cloud. Descriptive statistical analyses and regression analyses will be used to describe the determinants of the resilience scores of HSPs. Furthermore, we will use propensity score matching methods [45] to assess the effect of (im)mobilities on health and healthcare access and to check for covariate balance between (im)mobilities settings groups, and (non) recent access to HSPs These methods are applicable for any generalized linear model form (logistic, probit, log linear, Poisson) depending on the outcome measure’s distribution and the desired effect measure scale. Life-event analyses (sequence analysis and cox model for example) will be also used to analyse changes in health and mobility during life path [46].

Concerning secondary data (SARA and DHMIS), the health systems resilience analysis will be carried out in relation to their capacity to maintain the use of care by populations. We will rely on DHMIS data to conduct interrupted time series analyses (ITSA) or difference-in-differences methods (when control groups will be available) - using the main events identified during the exploratory phase as interruptions – to examine changes in health service utilisation indicators over time [47]. Like Odhiambo et al. [47], we will seek to collectively determine thresholds for each of the dimensions of our resilience scale (e.g. maintaining function as at least 80% of the 14 MNCH services maintained or improved their indicator values). We will rely on SARA survey data (both previously collected data and own data) to analyse changes in health service delivery preparedness over time and related those to the occurrence of CIEC. We will also use SARA survey data to generate explanatory variables that combine with our provider survey data to explain variation in resilience measures. These analyses will rely on the application of fixed-effects regression models. Statistical analyses will be performed with the R and RStudio software.

##### Triangulation

The analysis of qualitative and quantitative data will be articulated according to an integrated plan [38]; the data analyses from the qualitative phase (Phase 2) will enhance the questionnaires from the quantitative phase (Phase 3), and the data from the qualitative phase (Phase 4) will inform Phase 3 in more detail. The integration of quantitative and qualitative data will first help analyse the vulnerability of health systems and populations in the context of changes, events and risks, focusing on climate-induced environmental change and then provide a better understanding of the concept of resilience.

#### Ethics

Ethics approvals have been granted from the Institutional Review Board (IRB) of the BRAC James P Grant School of Public Health, BRAC University (ref: IRB-19 November’20–050) in Bangladesh and from the Comité National de Bioéthique in Haiti (ref: 2021–10). We will not collect biological data. We will ensure usual considerations concerning fieldwork ethics and research involving humans. However, in Haiti, given the complex socio-political context, an advisory committee including national researchers, civil society representatives and health system focal point from both selected sites, will be set up to discuss issues of health ethics principles, climate change [48] and violence, in particular. Besides, this committee will provide advice and guidance in data collection in difficult areas. If conditions for quantitative data collection become too challenging, a new protocol, involving only qualitative research and a maximum of online/telephonic qualitative interviews, will be proposed. It will include the qualitative phase described here (phase 2), followed by another qualitative phase (new phase 3). This updated phase 3, the design of which will depend on the results of phase 2, will establish a typology of events and individual, household and community strategies when facing the events, since the phase 2. If necessary and possible, a third qualitative phase following similar patterns will follow.

Participation to the study will be voluntary. We will inform participants about the research results’ intended publications, information on data use and sharing. We will also inform the participants about their right to withdraw at any time without consequences and about their right to refuse to answer. For the quantitative data collection, the consent form will be completed for all participants or those legally allowed to consent on their behalf. Because being underaged, the consent forms will be asked to the adolescents and their caregivers. Questions about children will be asked to their caregivers. We will interview participants in an adequate setting allowing confidentiality. We will replace the respondents’ names by ID codes that will not appear in the public databases. Once all data are imputed and ready to use, the key, associating names and ID codes, will be destroyed. For the other data collection, depending on the people interviewed and the local issues at stake, written or verbal consent will be systematically requested from participants. The usual ethical criteria for qualitative health research will be respected. Methodology concerning consents was approved by both ethics’ committees in Bangladesh and Haiti. A pilot study will assess the cultural sensitivity of some questions and adjustments will be decided with the local teams. Most of the sensitive questions have already been tested for the SARA/DHS surveys. Enumerators are trained to appropriate policy and practice concerning confidentiality, anonymity or acknowledgement of research participants. Feedback to participants on the research results as appropriate will be planned.

#### Knowledge transfer

Knowledge transfer will take place throughout the project, in close collaboration with stakeholders. Support will be offered in each of the two countries to develop and implement a knowledge transfer plan. These plans will be adjusted throughout the project according to its evolution. The plans will (at least) include the presentation of results through research snapshots and policy briefs [49] in English and national languages. We will also disseminate our finding at the national, regional and local Health Authorities through deliberative workshops [50] informal meetings, presentations, and policy briefs, which will have been reviewed by stakeholders for content and presentations [49] and also to other countries struggling with climate change, through policy briefs as well as through international and regional networks. Scientific communication is planned through publications and presentations at national and international conferences. Part of the material, such as research snapshots, policy briefs, evaluation protocols, videos and evaluation findings will be publicly available (after publication for any results dissemination) through the project website.

Participatory visual anthropology will also be part of our knowledge transfer strategy. According to Baumann’s methodology [51], we will facilitate the creation of participatory videos in different communities. These videos will visually represent the complex issues of migrant access to healthcare, advocate for an embodied and bottom-up approach to healthcare issues and help communities to dialogue with public decision-makers. We will develop a Memorandum of Understanding with project participants to include project objectives based on stakeholders and consent to film and/or be filmed. In addition, a workshop will be organised between the viewing of the edited film and the broadcast to obtain consent. Consent will also be the subject of in-depth discussions on image rights, broadcasting rights and signing of a form by participants and researchers. A specific research protocol for these videos will be published.

## Discussion

Global changes, such as climate changes and variability, (im)mobilities and pandemics, are part of the most prominent contemporary health challenges. However, very little has been done to study health systems resilience and climate change [52]. Due to these systems’ complex nature and mechanisms of these systems, an interdisciplinary approach is needed [52]. Here, we present, an innovative and integrative conceptual framework associated with an interdisciplinary mixed-method research design.

We based our conceptual framework on two complementary frameworks: one on healthcare access and the other on the resilience process. The Levesque’s Conceptual Framework of Access to Health [15] advantages reside in its two-sides approach, which focuses on the interactions between healthcare service providers and populations, i.e. between demand and supply. More specifically, it illustrates the interactions between the five dimensions related to the health system (approachability, acceptability, availability and accommodation, affordability et appropriateness) and the five capacities related to individuals or households (abilities to perceive, to seek to reach to pay and to engage) during the care pathways. According to a scoping review on the use of Levesque’s framework, since its conceptualization in 2013, it has been used successfully in empirical researches in various healthcare services and settings. It allows “to comprehensively assess the complex and dynamic process of healthcare access” [53]. In the context of universal health coverage (UHC), we consider that access to care is the proximal outcome of health systems’ resilience but more is needed considering the mechanisms of the resilience processes. For this reason, we decided to combine the Levesque’s framework [15] to the FCDO framework [16] to include a dynamic approach of healthcare access while considering the context, the disturbance, the capacity to deal with disturbance, and the reaction to it. This choice allows an innovative, integrative and systemic approach of resilience, while considering contexts and vulnerabilities [10–12]. We chose an interdisciplinary mixed-method exploratory protocol for the framework operationalisation [38]. This multi-step protocol optimises primary and secondary data use, for a transversal and longitudinal approach within a 2-year timeframe in both countries. The multi-sites qualitative and quantitative data collection, with a sequential exploratory/explanatory design, permits an understanding and adjustments to local contexts. Spatial analysis, multivariable and multi-level analyses (individual, household, health provider, cluster, and country levels) will be conducted to understand local context variations.

Due to events uncertainty in the study sites in such a short time-frame and the long span of the Covid-19 pandemic, we ruled out a second data collection for the longitudinal approach. We preferred an approach based on secondary data, supplemented with our primary data.

During the elaboration of the conceptual framework, we encountered several difficulties. First, we focused on climate change, as it is often overlooked, yet non negligeable [52, 54]. The link between climate change and climate events is a complex and controversial topic [55–57]. However, there is growing evidence that extreme weather events are amplified by climate change, as shown by the development of the climate attribution field [58, 59]. For this reason, we decided to focus on climate-related events, such as drought, flood and cyclone. Besides, because other changes and events might be closely intertwined with climate, our methodology allows us to include other global and local events, changes and risks impacting local health systems and populations [54]. We will list, for each site, events, changes and risks affecting locally populations and HSPs. As our research is taking place in the context of the COVID-19 pandemic, we will also be attentive to the relative perception of this shock compared to others for health systems.

Second, as a result of our preliminary literature review, no consensus amongst scholars on ‘climate-induced migration’ definitions, conceptual approaches or frameworks are to be found in the literature [60, 61]. This is mostly due to the complex and indirect causal links between climate change and mobility. Indeed, Homer-Dixon [62] states that “isolating individual causal agents of population movement as entirely environmental inappropriately emphasizes one cause amongst an array of overlapping and interconnected processes” and for Black [63] “conceptualizing environmental change as a primary cause of displacement is unhelpful, intellectually unsound, and unnecessary”. Rather than focusing on the nexus climate change-migration [64, 65] which would have been too complex to define and could be controversial, we have preferred to include all migration forms. Indeed, all types of migration might influence health systems - whether at the HSP level or at the level of individuals, families and communities.

Data collection in times of uncertainty is complicated to plan. We are working in two countries with different contexts in a time of pandemic. Currently, fieldwork in Haiti is extremely complicated and costly for security and access reasons. This is leading us to take into account the ethics of research in a context of repression and reduction of individual freedoms and human rights. We decide to put in place additional measures, such as the creation of a local advisory committee on health ethics principles in the context of climate change [48] and violence that will be active throughout the project, over and above what is usually required. Secondary data availability is a challenge for country comparison at the local level. Thus, we will focus mostly on longitudinal and transversal analyses inside each country, and country comparison will be made when possible.

## Conclusion and expected outcomes

The CLIMHB project aims to provide an evidence base for research and policy development for health systems resilience in the context of global changes, in particular climate-related events, population mobility, pandemics and their intersections. This interdisciplinary project’s originality lies in its combined approach to the health system’s process and output in the context of global changes and its local impacts, i.e. access to care and resilience, at both institutional and population levels. This project will contribute to the developing field of research on LMIC health systems and climate change, as prescribed by Sustainable Development Goals 13 (climate change) and 3 (health) [66]. Besides, this project will suggest operational solutions and lessons learned from decision-makers from the national level to the community level. Finally, it will support communities’ discussions around health services access, health practices and overall communities’ social and political organisation.

## Supplementary Information

**Additional file 1.** Quantitative tool: quantitative questionnaire for the population. QuantiPop.

**Additional file 2.** Quantitative tool: questionnaire for the health service providers. QuantiHSP.

**Additional file 3.** Qualitative tool: interview guide for the population. QualPop.

**Additional file 4.** Qualitative tool: interview guide for the health service providers. QualHSP.

**Additional file 5.**

**Additional file 6.**

**Additional file 7.**

**Additional file 8.**

**Additional file 9.**

## Data Availability

Secondary data are available on the DHS website: https://dhsprogram.com/data/available-datasets.cfm

## Abbreviations

CIEC: Climate-induced environmental change
CLIMHB project: Climate Change, Migration and Health Systems Resilience in Haiti and Bangladesh project
DFID: Department for International Development
DHMIS: District Health Management Information Systems
FCDO: (UK) Foreign, Commonwealth and Development Office
HSP: Health service provider/healthcare service provider
HSR: Health System Resilience/Healthcare System resilience
ITSA: Interrupted Time Series Analyses
LMIC: Low- and middle-income countries
MNCH: Maternal, Newborn and Child Health
NGO: Non-Governmental Organisation
SARA: Service Availability and Readiness Assessment
SDG: Sustainable Development Goal
UHC: Universal Health Cover
WHO: World Health Organization
