# Supplementary material for: Healthcare system resilience in Bangladesh and Haiti in times of global changes (climate-related events, migration and Covid-19): an interdisciplinary mixed method research protocol
Source: BMC Health Serv Res. 2022 Mar 15;22:340. doi: 10.1186/s12913-021-07294-3 (PMC8921708; doi:10.1186/s12913-021-07294-3)
Supplement: Supplementary file 3 — Additional file 3. Qualitative tool: interview guide for the population. QualPop. [file 12913_2021_7294_MOESM3_ESM.docx]

# POPULATION COMPONENT

# Qualitative Data Collection (Phase 2)

## Focus Group Discussions (FGDs) per site: 9

## Populations (3), community-based groups (3) and identified patients (3)

1. {Visual Mapping Exercise} Listing local forms of mobility (section 1 of the CF): frequency, form, duration, intensity, socio-economic drivers, etc.

- Is mobility important in [name of locality]? Are there different sorts of migrants within [name of locality]? Where do they come from? What are their motivations to come here? How long do they stay? Who within the family usually migrates (male individual, female individual, entire household = n)?
- Were there episodes of out-migration within [name of locality]? What were the reasons? Where did people go? When did they return?

Are some long-term residents willing but not able to move out of [name of locality]? What are the reasons?

1. List of most frequented healthcare service providers in the community

- Where/ To whom do people from the community most frequently go to when they seek medical consultation or health treatment?

1. {Visual Mapping Exercise} How do the following abilities influence {local forms of mobility}, starting with the most frequent one:

Particularly, how do the {dimensions below} influence (or not) mobility decisions?

- - Assets: …
  - Social Capital: …
  - Social Support: …
  - Caregiver Support: …
  - Health Insurance: …
  - Culture and Values: …
  - Gender: …
  - Living environments: …
  - Transport and mobility: ….
  - Health Literacy: …
  - Health beliefs: ….
  - Information: ….
  - Empowerment: …

*(If other social dimensions come up during discussions, include them on the list)*

These determinants are part of these dimensions (discussion around the determinants to classify into the following dimensions according to local contexts)

- to perceive healthcare needs

- to seek healthcare

- to reach healthcare service providers

- to pay health care delivery

- to engage in health prevention and healthcare promotion

1. Based on the most frequent forms of local migration, starting with the most frequent one, explain the strategies set up to enhance access to care?

- How do [name of aforementioned mobility type] migrant get access to healthcare in the [name of locality]? Could you give specific examples on how these migrants use healthcare when needed? Do they develop alternative healthcare pathways?

1. What have been the most disruptive events in the last X years?

- Environmental events?
- Socio-political events?

Socio-demographics of respondents

Number of respondents:

Age/gender/occupation/place of birth/place of living/ relationships to each other (in the group discussion) if any/ have migrated/have a migrant in their household/ etc.
