# Supplementary material for: Healthcare system resilience in Bangladesh and Haiti in times of global changes (climate-related events, migration and Covid-19): an interdisciplinary mixed method research protocol
Source: BMC Health Serv Res. 2022 Mar 15;22:340. doi: 10.1186/s12913-021-07294-3 (PMC8921708; doi:10.1186/s12913-021-07294-3)
Supplement: Supplementary file 4 — Additional file 4. Qualitative tool: interview guide for the health service providers. QualHSP. [file 12913_2021_7294_MOESM4_ESM.docx]

# HEALTHCARE SERVICE PROVIDER COMPONENT

QUALITATIVE TOOL

## **Phase 2: Qualitative Data Collection**

*Qualitative interviews with the heads of the 10 local health facilities per site; and with decision-makers at the local, regional and national levels*

*Preliminary work: Enumeration of the events impacting negatively or positively the organisational routine of the HSP*

*Ranking of those events, from the most to the least important one in terms of impact.*

Themes addressed (discussions will be broader at national level):

1. **Based on the most important selected events, starting with the most important one, explain the effects on the system routine (the 10 selected dimensions of HS); (section 5.1 of CF)**

Could you tell me how this event disrupted your normal functioning, routines and habits? Could you give me specific, concrete examples of these disruptions?

These disruptions may concern (Investigator: Record examples for each of these 10 dimensions or other dimensions.) : «*Governance*»; « *intervention level* »; «*workforce*»; « *Cultural and social values* »; «*Finance*»; « *planning and supporting guidance* »; « *systems specificities* » « *Health sector management* » « *information systems* »; «*Context and security* »

1. **Based on the most important selected events, starting with the most important one, explain the actions/strategies initiated to deal with these disruptions (in the 10 dimensions of the HS); (section 5.2 of CF on adaptive capacities)**

For each of the disturbances you just named, could you explain to me how you reacted and coped? What actions did you take to deal with them, to adapt to this event? Could you give me specific examples of how you have acted in practice?

These actions to cope may concern the 10 dimensions or others.

1. **Based on the most important selected events, starting with the most important one, what are the impacts for the determinants of service use, (sections 6 and 3 of CF)**
2. *Supply Dimensions:*
   1. *Approachability: Can you please explain us in detail, what is being done to improve community awareness of existing healthcare services offered by (Name of Healthcare Center)?*
   2. *Acceptability: How does language, culture or religion (of health providers and your patient) influences the healthcare that is provided in (Name of Healthcare Center)?*
   3. *Availability: How does (Name of Healthcare Center) ensures that essential healthcare services (a list will be done by country) are always available to clients/patients?*
   4. *Affordability: What do you think about the cost of basic (PHC) healthcare services offered in (Name of Healthcare Center)?*

*Appropriateness: How does (Name of Healthcare Center) makes sure that the patients sufficiently understands health advises and instructions before they leave the facility?*

1. *Demand Dimensions:*
   1. *To Perceive: In your opinion how much does the community trust the information and services provided by (Name of Healthcare Center)?*
   2. *Seek: Can you describe any instance/s where patients expressed concern on the need to make the health services in (Name of Healthcare Center) more culturally sensitive?*
   3. *Reach: How does the distance and travel time from the patient's residence to (Name of Healthcare Center) affects they ability to reach, the frequency of their visit or follow up?*
   4. *Pay: What do you think about the ability to pay for patients who go to (Name of Healthcare Center)?*

*Engage: How have patients responded to your (health providers) efforts give them adequate information and involvement in decisions regarding their treatment options or care?*

1. **Based on the most important selected events, starting with the most important one, what are the impacts for the use of healthcare services (sections 7 of CF, between 3 and 6)**

Overall, after thinking about each of the 10 determinants of access to care from a health system and population perspective, for each of the important events, what would you say about these impacts on the use of services in your health center?

1. **Based on most important events, starting with the most important one, what is the overall perception of the resilience level (see protocol definition : i.e the capacity to maintain access to care of their health facility (or of the system as a whole) in the perspective to reduce/maintain/improve access to care (section 9 of CF)**

Generally speaking, after thinking about the most important event and its impact on the use of health services; what do you think of the resilience of your health center in this particular context? Did your health center improve, recover, deteriorate or collapse? Could you provide details and examples to illustrate your assessment of this level of resilience?

1. **Based on the most important selected events, starting with the most important one, what is the final perception of the effects on the health of populations (section 9 of CF)**

Finally, with regard to the impact on the use of services and the resilience of your health center in the face of the most important event, what do you think of the health status of the population in your health area in general? How do you rate it overall? What do you think of the situation for their mental health, their living conditions or their economic situation in the context of this event and once they have been in your health centre.

Socio-demographics of respondents:

- *Age : date*
- *Gender : M/W*
- *Highest diploma*
- *Type of jobs : responsible/non responsible*
- *Duration in employment : years*
- *Duration of presence in this health facility : years*
