# Supplementary figures and images for: Healthcare system resilience in Bangladesh and Haiti in times of global changes (climate-related events, migration and Covid-19): an interdisciplinary mixed method research protocol
Source: BMC Health Serv Res. 2022 Mar 15;22:340. doi: 10.1186/s12913-021-07294-3 (PMC8921708; doi:10.1186/s12913-021-07294-3)

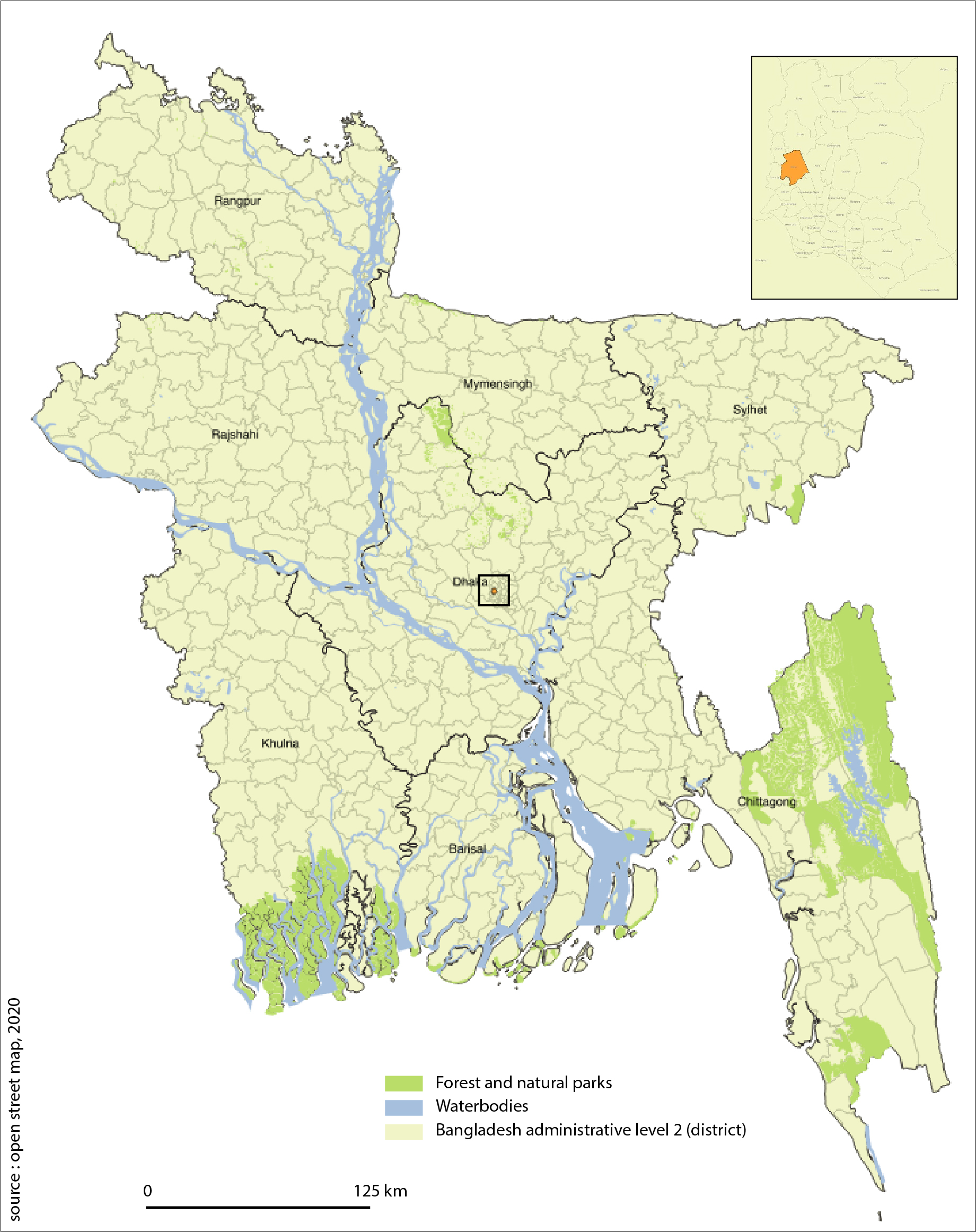

Supplement: Supplementary file 9 — Additional file 9. [file 12913_2021_7294_MOESM9_ESM.jpeg]
